# Supplementary material for: Maternally inherited genetic variants of CADPS2 are present in Autism Spectrum Disorders and Intellectual Disability patients
Source: EMBO Mol Med. 2014 Apr 14;6(6):795–809. doi: 10.1002/emmm.201303235 (PMC4203356; doi:10.1002/emmm.201303235)
Supplement: Supplementary file 1 — Supplementary Figure S1 [file emmm0006-0795-sd1.pdf]

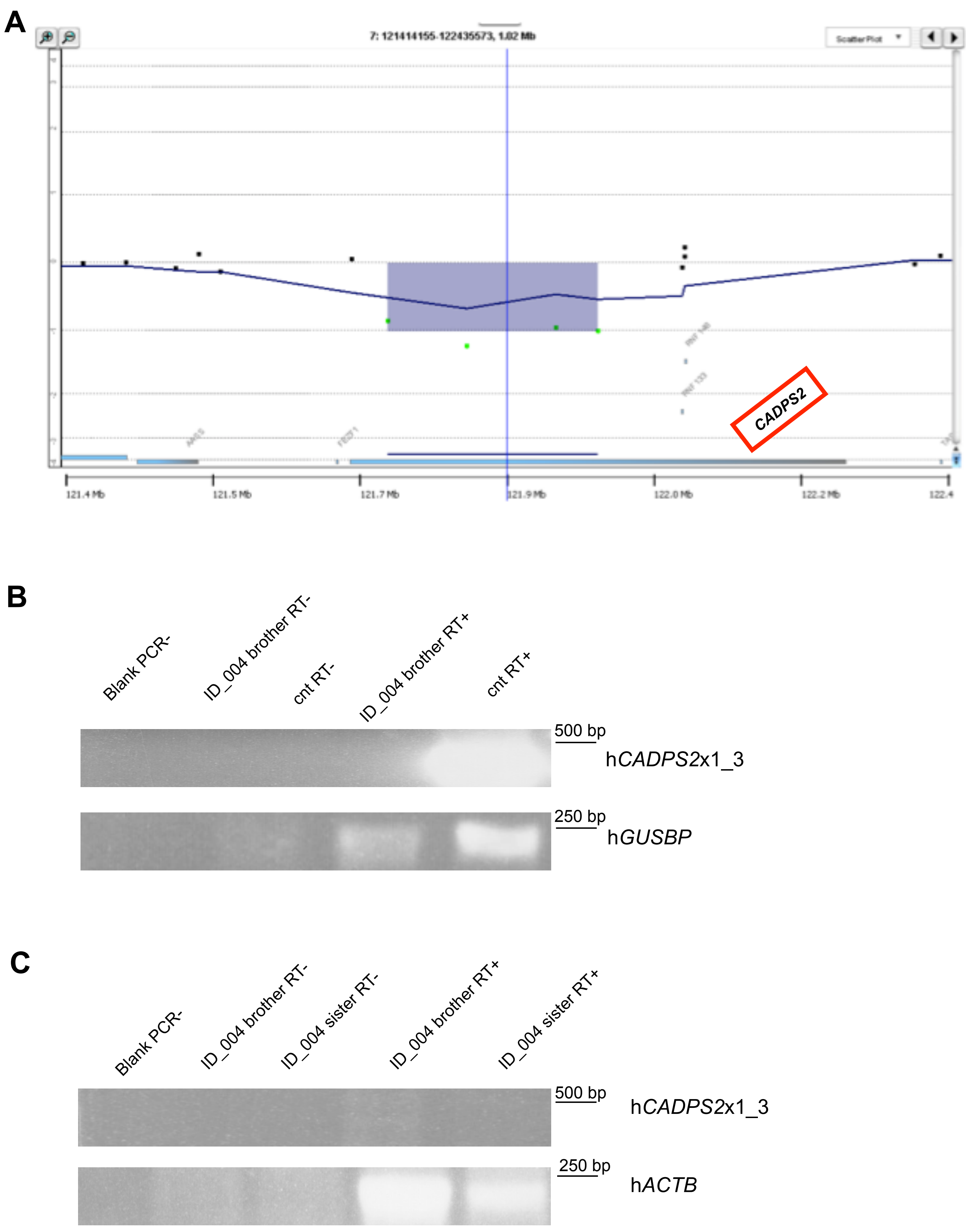
A
7: 121414155-122435573, 1.02 Mb
CADPS2
121.4 Mb
121.5 Mb
121.7 Mb
121.9 Mb
122.0 Mb
122.2 Mb
122.4
B
Blank PCR-
ID_004 brother RT-
cnt RT-
ID_004 brother RT+
cnt RT+
500 bp
hCADPS2x1_3
250 bp
hGUSBP
C
Blank PCR-
ID_004 brother RT-
ID_004 sister RT-
ID_004 brother RT+
ID_004 sister RT+
500 bp
hCADPS2x1_3
250 bp
hACTB

Figure S1: (A) Array-CGH profile centered on chromosome 7q31.32 deletion. This view is derived from Genomic Workbench software (Agilent Technologies), which visualizes normal and deleted oligonucleotides as black and green filled circles, respectively, and genes as light blue bars. CADPS2 gene is highlighted by a red box. Numbers indicate genomic positions (hg18); (B,C) RT_PCR for *CADPS2* expression in ID_004 patients carrying the deletion vs a control: (B) upper lane, *CADPS2* expression (primer mapping in exon 1 and exon 3) from patient ID_004.01 and from a control cDNA, lower lane, *GUSBP* expression (control gene); (C) upper lane, *CADPS2* expression in the two ID_004 sibs, lower lane, *ACTB* expression (control gene). Rt+= reverse transcription in presence of the RT enzyme, RT-= reverse transcription in absence of the RT enzyme.
