# Supplementary material for: Maternally inherited genetic variants of CADPS2 are present in Autism Spectrum Disorders and Intellectual Disability patients
Source: EMBO Mol Med. 2014 Apr 14;6(6):795–809. doi: 10.1002/emmm.201303235 (PMC4203356; doi:10.1002/emmm.201303235)
Supplement: Supplementary file 2 — Supplementary Figure S2 [file emmm0006-0795-sd2.pdf]

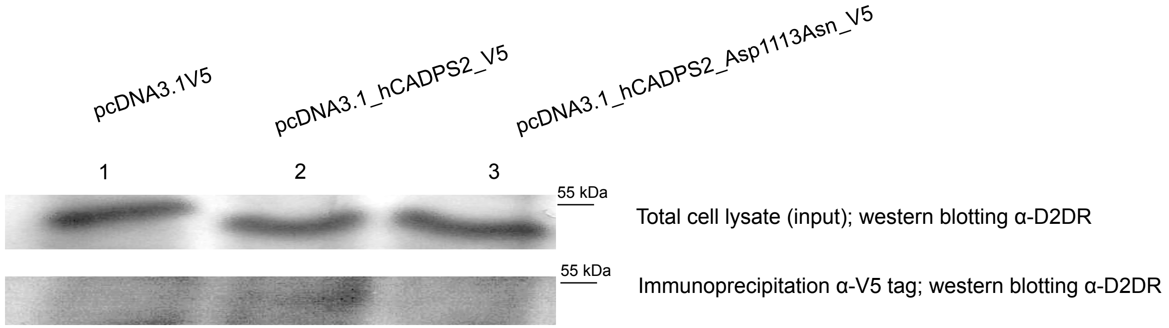

Figure S2: Western blot analysis of SHSY5Y cell lysates transfected with empty V5 (lane 1), CADPS2_wtV5 (lane 2), CADPS2_D1113N_V5 (lane 3) vectors and immunoprecipitated with anti-V5 antibody and probed with anti-D2DR antibody: upper panel, input showing the equal amount of endogenous D2DR; lower panel, western blot showing the co-immunoprecipitated amount of D2DR receptor when the input is immunoprecipitated with anti-V5 antibody: only in lane 2 (CADPS2_wt_V5) it is visible the band corresponding to D2DR.
