# Supplementary material for: Maternally inherited genetic variants of CADPS2 are present in Autism Spectrum Disorders and Intellectual Disability patients
Source: EMBO Mol Med. 2014 Apr 14;6(6):795–809. doi: 10.1002/emmm.201303235 (PMC4203356; doi:10.1002/emmm.201303235)
Supplement: Supplementary file 3 — Supplementary Table S1 [file emmm0006-0795-sd3.pdf]

Table S1: Additional CNV identified in the family ID_004

	Position (hg18) Kb	Reported in DGV	Proband	Sister	Father
dup9p24.3	601 – 883	Yes	Yes	Yes	No
del17q23.2	56,464 – 56,682	Yes	Yes	No	Yes
