# Supplementary material for: Maternally inherited genetic variants of CADPS2 are present in Autism Spectrum Disorders and Intellectual Disability patients
Source: EMBO Mol Med. 2014 Apr 14;6(6):795–809. doi: 10.1002/emmm.201303235 (PMC4203356; doi:10.1002/emmm.201303235)
Supplement: Supplementary file 4 — Supplementary Table S2 [file emmm0006-0795-sd4.pdf]

Table S2: Clinical characteristics of ASD (a) and ID (b) patients included in the study.

a) ASD

			**Sex**		**Intellectual disability**				
		**Total**	**Male**	**Female**	**severe 20<IQ<34**	**moderate 35<IQ<49**	**mild 50<IQ<69**	**normal IQ≥70**	**Unknown**
**ASD sample**	SM	72	58	14	1	10	25	33	3
	Catania	22	21	1	2	3	2	4	11*
	**Total**	**94**	**79**	**15**	**3**	**13**	**27**	37	14

* 10 were assessed by PEP-R (QS score ranging from 16 to 55)

b)

**Sex**		**Intellectual Disability**			**Epilepsy**	**ASD features**		
**Male**	**Female**	**Severe 20<IQ<34**	**Moderate 35<IQ<49**	**Mild 50<IQ<69**		**Impairments in verbal communication and social interaction; stereotyped behaviors**	**Social impairment**	**Stereotyped behaviors**
22	15	12	14	11	17	3	5	7
**Total= 37**						**Total=15**		
