# Supplementary material for: Maternally inherited genetic variants of CADPS2 are present in Autism Spectrum Disorders and Intellectual Disability patients
Source: EMBO Mol Med. 2014 Apr 14;6(6):795–809. doi: 10.1002/emmm.201303235 (PMC4203356; doi:10.1002/emmm.201303235)
Supplement: Supplementary file 5 — Supplementary Table S3 [file emmm0006-0795-sd5.pdf]

Table S3: Data for the two *CADPS2* DMRs from blood DNA. Data are shown represent the mean±standard deviations values.

| **DMR ID** | **GENE** | **ID (n=16)** | **CNT (n=18)** |
|---|---|---|---|
| Intron1CpG_15 | *CADPS2* | 0.300±0.092 | 0.333±0.083 |
| Intron1CpG_16 | | 0.386±0.052 | 0.303±0.050 |
| SQNM | *IGF2* | 0.273±0.051 | 0.297±0.021 |
