# Supplementary material for: Maternally inherited genetic variants of CADPS2 are present in Autism Spectrum Disorders and Intellectual Disability patients
Source: EMBO Mol Med. 2014 Apr 14;6(6):795–809. doi: 10.1002/emmm.201303235 (PMC4203356; doi:10.1002/emmm.201303235)
Supplement: Supplementary file 6 — Supplementary Table S4 & Table S5 [file emmm0006-0795-sd6.pdf]

Table S4: Primer sequences for MS analysis and CpG content details for each amplicons.

a) Human *CADPS2*

Amplicon position from CDS	Target length (bp)	Primer Forward	Primer Reverse	Strand	CpGs contained	CpGs analyzable
-1421; -927 (a)[a]	495	GAAAGTGGTTTGAAAAAGTTAAAATTG	ACAAAATAATACTAATTCAACTCAAACA	Forward	12	10
-911; -486 (b)	426	TTTGTTTTGAGTTTGTAGGATTAGAA	CCTAAAACCCTAACACACAATTTACA	Forward	22	13
-515; -112 (c)	404	GAAAGGAAAATTGGTTAGGGT	CCTTTACAAACTATATACTAAAACTCCAAA	Reverse	41	31
+550; +973 (d)	424	GGGTTGGTAGGAGTTGTTAGTTTATT	CAAACCTACCCAAACCAAAAATTAC	Forward	17	9

a. (a) to (d) refers to positions indicated in Figure 3a.

Table S5: *Cadps2* coding SNPs in two mouse strains.

SNP ID	Type of change	FVB genotype	C57 genotype
rs33756726	G>A	**G/G**	**A/A**
rs30999238	C>T	T/T	T/T
